# New Labdanes with Antimicrobial and Acaricidal Activity: Terpenes of *Callitris* and *Widdringtonia* (Cupressaceae)

**DOI:** 10.3390/antibiotics9040173

**Published:** 2020-04-11

**Authors:** Nicholas J. Sadgrove, Haytham Senbill, Ben-Erik Van Wyk, Ben W. Greatrex

**Affiliations:** 1 School of Science and Technology, University of New England, Armidale, NSW 2351, Australia; ben.greatrex@une.edu.au; 2 Department of Botany and Plant Biotechnology, University of Johannesburg, Johannesburg 2006, South Africa; bevanwyk@uj.ac.za; 3 Jodrell Laboratory, Royal Botanic Gardens, Kew, Richmond, Surrey TW9 3AB, UK; 4 Department of Entomology, Faculty of Agriculture, Assam Agricultural University, Jorhat, Assam 785013, India; haytham.senbill@alexu.edu.eg; 5 Department of Applied Entomology & Zoology, Faculty of Agriculture, Alexandria University, Alexandria 21545, Egypt

**Keywords:** *Widdringtonia*, *Callitris*, diterpene, cedar, essential oil, antimicrobial, NMR

## Abstract

In spite of the evidence for antimicrobial and acaricidal effects in ethnobotanical reports of *Callitris* and *Widdringtonia*, the diterpene acids from *Widdringtonia* have never been described and no comparison to the Australian clade sister genus *Callitris* has been made. The critically endangered South African Clanwilliam cedar, *Widdringtonia wallichii (syn. W. cedarbergensis)*, of the Cederberg Mountains was once prized for its enduring fragrant timbers and an essential oil that gives an aroma comparable to better known Mediterranean cedars, predominantly comprised by widdrol, cedrol, and thujopsene. In South Africa, two other ‘cedars’ are known, which are called *W. nodiflora* and *W. schwarzii*, but, until now, their chemical similarity to *W. wallichii* has not been investigated. Much like *Widdringtonia*, *Callitris* was once prized for its termite resistant timbers and an ‘earthy’ essential oil, but predominantly guaiol. The current study demonstrates that the essential oils were similar across all three species of *Widdringtonia* and two known non-volatile diterpene acids were identified in leaves: the pimaradiene sandaracopimaric acid (**1**) and the labdane *Z*-communic acid (**2**) with a lower yield of the *E*-isomer (**3**). Additionally, in the leaves of the three species, the structures of five new antimicrobial labdanes were assigned: 12-hydroxy-8*R*,17-epoxy-isocommunic acid (**4**), 8*S*-formyl-isocommunic acid (**5**), 8*R*,17-epoxy-isocommunic acid (**6**), 8R-17*R*-epoxy-*E*-communic acid (**7**), and 8*R*-17-epoxy-*E*-communic acid (**8**). Australian *Callitris columellaris (syn. C. glaucophylla)* also produced **1** and its isomer isopimaric acid, pisiferal (**9**), and pisiferic acid (**10**) from its leaves. *Callitris endlicheri* (Parl.) F.M.Bailey yielded isoozic acid (**11**) as the only major diterpene. Diterpenes **4**–**6**, pisiferic acid (**10**), spathulenol, and guaiol (**12**) demonstrated antimicrobial and acaricidal activity.

## 1. Introduction

Southern African *Widdringtonia* were once prized for their timbers and have been heavily exploited over the course of more than a century. The most pronounced impact has been on the now critically endangered *W. wallichii* Endl. ex Carrière (=*W. cedarbergensis* J.A.Marsh) [1], which was favoured for its durable fragrant timber [2,3]. Unlike other species in the genus, it does not re-sprout after fire [3,4] and so its conservation status is now critical.

The earliest chemical study of *Widdringtonia* had allegedly investigated the ‘five’ species that are endemic to South Africa [5], but, due to taxonomic revisions, the earlier five [6] have been reduced to three. Thus, today it is unclear specifically which species were examined since the provenances of their collections were not recorded. Nevertheless, one of the three South African *Widdringtonia* was examined, which includes *W. wallichii* (widely referred to in the historical and contemporary literature by the illegitimate name *W. cedarbergensis*), *W. nodiflora* (L.) E.Powrie, and *W. schwarzii* (Marloth) Mast. The most widespread species is *W. nodiflora*, which is previously known under at least 18 synonyms [7] including the more common synonyms *W. cupressoides* (L.) Endl., *W. juniperoides* (L.) Endl., and *W. dracomontana* Stapf. In this paper, we consistently follow the recommended taxonomic concepts and nomenclature as given in “The Plant List” (http://www.theplantlist.org/) and the now finalized international plant name index (IPNI: https://www.ipni.org/: 2 March 2020).

**Figure 1:**
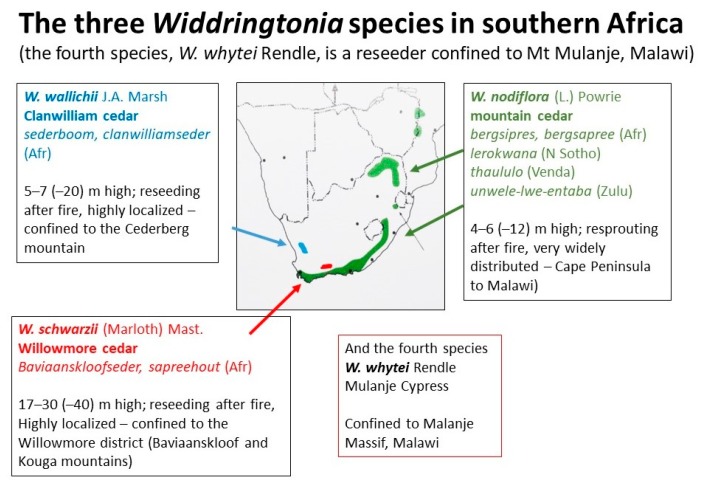
Distribution of the three species of *Widdringtonia* in South Africa.

The study of solvent extracted material from heartwood of various taxa in *Widdringtonia* by Erdtman and Thomas [5] described several sesquiterpenes. In that study, the identity of ‘Acid III’ was later revealed to be cuparenic acid [8] and ‘widdrene II’ was cuparene [9], which is evident in hydro-distilled essential oils [10,11]. The identity of ‘widdrene’ was later revealed to be thujopsene, ‘widdrenal’ as thujopsenal and ‘widdrenic acid’ as hinokiic acid [12]. Only ‘widdrol’ was understood to be new and so the name has been accepted [9]. Cedrol is another of the important sesquiterpenes, which gives an olfactory likeness to the Mediterranean cedars [13]. This is evidently of etymological importance for the common but outdated synonym *W. cedarbergensis* (correctly *W. wallichii*), which is endemic to the Cederberg mountains in Clanwilliam (South Africa) (Figure 1).

South African *Widdingtonia* has long been recognised as closely alligned with Australian *Callitris*, with the two described as clade sisters [14]. Due to the extreme disjunction between the two genera, it has been suggested that they form part of a relict group with many extinct members [15]. Unlike *Widdringtonia*, Australian *Callitris* is still widespread despite the historical popularity of the timbers for their termite resistance. The most common species are *C. columellaris* F. Muell (coastal cypress pine), *C. endlicheri* (Parl.) F.M.Bailey (black cypress), *C. glaucophylla* Joy Thomps. & L.A.S.Johnson (white cypress) and *C. intratropica* (R.T.Baker & H.G.Sm.) Silba (blue cypress). The latter two, *C. glaucophylla* and *C. intratropica*, are not recognized as distinct outside of Australia, and are classified as synonyms of *C. columellaris* by The Plant List. Nevertheless, chemical characterization of various extractives from these geographically segregated taxa demonstrates consistent chemical divergence [16]. In the current study, specimens of *C. glaucophylla* are referred to as *C. columellaris* to remain consistent with the international recommendation.

Essential oils from timbers of *Callitris* predominantly consist of guaiol [17], along with other sesquiterpenes such as azulenes [18], which are responsible for the blue colour of material referred to as *C. intratropica* essential oil (guaiazulene, chamazulene). Costols [18] and ƴ-lactones [19] are also present and aid termite resistance [20]. Guaiol and azulenes have higher relative abundances in the essential oils, while costols and ƴ-lactones are more strongly represented in solvent extracts [20] due to their higher boiling points. A recent study of *Callitris* spp. also reported diterpene acids using mass spectrometry on extracts from the resins [21].

As with all members of Cupressaceae, leaf essential oils from *Callitris* and *Widdringtonia* are significantly different from heartwoods [10,16] with monoterpenes predominating in leaf oils and sesquiterpenes predominating in heartwood timber oils. A study on *Callitris* leaves by gas chromatography reported only one diterpene, pisiferal [16], but the major non-volatile diterpenes from *Callitris* have remained hitherto unidentified, as is the case for *Widdringtonia*. Both *Widdringtonia* and *Callitris* have been extensively milled for timber, which resulted in continued interest in valorisation of the saw dust waste [11,22]. Strangely, no similar interest has been expressed for leaves that are also a by-product of the logging process. In the current study, we have investigated the chemistry of leaves and, in some cases, we have contrasted between leaves and branch/twig pulp. Traditional use reports describe topical therapeutic applications of leaf extracts of *C. columellaris* and cone extracts (clear hard gum) of *Widdringtonia* spp. for ailments consistent with microbial or parasitic (ectoparasitic) infection [16,23]. Thus, isolated compounds from *Callitris* and *Widdringtonia* have also been examined for antimicrobial and acaricidal activity, as knowledge in the area is currently lacking [22].

## 2. Results and Discussion

### 2.1. Chemistry of South African Widdringtonia

For reasons of conservation, only leaves and branches (two-inch circumference) of *Widdringtonia* were extracted in the present study, as heartwood harvesting is destructive. A series of known (**1**–**3**) and new labdane derivatives (**4**–**8**) have been identified in extracts from leaves and other aerial parts (Table 1), along with volatile terpenes consistent with those identified in earlier phytochemical studies on essential oils from *Widdringtonia* (Table 2). The overwhelmingly higher representation of diterpenes in the extracts of leaves is surprising, but consistent across all specimens and species investigated in *Widdringtonia*. Since diterpenes were not isolated in the earlier studies that focused on heartwood, it is likely that the diterpenes are restricted to branching parts and sesquiterpenes are obtained in higher yield in the heartwood.

The most abundant diterpenes are already well known from Cupressaceae with sandaracopimaric acid (**1**) being the dominant terpene in our extracts, which is followed by *Z*-communic acid (**2**) (Table 1). The name sandaracopimaric acid has its etymology in the product ‘sandarac’, historically derived from the Moroccan species *Tetraclinis articulata* (Vahl) Mast., which is another member of Cupressaceae [24]. The communic acid isomers are evidently familiar with the fruits of *Juniperus communis* L. [25], which is popularly known for its essential oil wherein the acids are absent due to higher boiling points.

**Figure 2:**
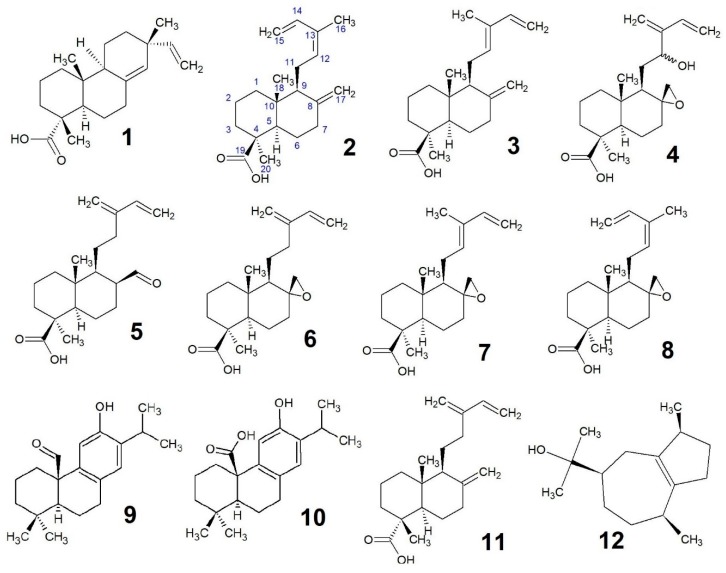
Structures of compounds **1**–**12**, Sandaracopimaric acid (**1**), *Z*-communic acid (**2**), *E*-communic acid (**3**), 12-hydroxy-8*R*,17-epoxy-isocommunic acid (**4**), 8*S*-formyl-isocommunic acid (**5**), 8*R*,17-epoxy-isocommunic acid (**6**), 8*R*-17-epoxy-*E*-communic acid (**7**), 8*R*-17-epoxy-*Z*-communic acid (**8**), pisiferal (**9**), pisiferic acid (**10**), isoozic acid (**11**), and guaiol (**12**).

The other lesser abundant diterpenes (**4**–**8**) (Nuclear Magnetic Resonance (NMR) data, Table 3 and Table 4) are undescribed and, as far as we can tell, restricted to *Widdringtonia*. The compounds were assigned as structural analogues of isocommunic and communic acids (Figure 2), differing by oxidation at either the C8 position or C12 on the branching chain moiety. The prevailing characteristic of these new diterpenes is the spiroepoxy at C8, which is evident in all except for the aldehyde (**5**).

In most plant parts, the spiroepoxy diterpenes were of low relative abundance, except for in the cones of *W. nodiflora*, which were richer in the new diterpenes as compared to 1 and 2. Furthermore, the cones yielded over 1% *w/w* spathulenol in flash chromatography. Spathulenol (Table 5) is an antimicrobial sesquiterpene [26] that demonstrates enhanced activity against skin pathogens when encapsulated (Table 6). An encapsulation effect similar to that derived from inclusion in the chemically diverse gum that exudes from the cones. Spathulenol is also present at lower concentrations in the essential oils of other plant parts (Table 5). The pronounced chemical difference of the cones compared to other plant parts provides the first insight into why these are chosen above other organs in therapeutic applications consistent with antimicrobial outcomes. Antimicrobial testing provided further testimony to this (Table 6). See Section 2.3 for more details.

Essential oils from leaves and stem pulp (Table 5) of *W. nodiflora* and *W. schwartzii* were chemically similar to essential oils from aerial parts of *W. wallichii* described in other studies [10]. While the essential oils of branches displayed high chemical consistency with that reported for the heartwood, the yields obtained by us were still lower than the reported yields in heartwood.

### 2.2. Chemistry of Callitris

Essential oils from leaves of the two species of *Callitris* sampled in the current study (*C. endlicheri* and *C. columellaris*) have been previously characterised [16,27]. Furthermore, timber essential oils and solvent extracts have also been studied [18]. Thus, in the current study, an examination of solvent extracted (non-volatile) components from the leaves was undertaken to gratify this neglected area of research in order to make a comparison to *Widdringtonia*. Unlike *Widdringtonia*, interspecific differences in the Genus were pronounced. Major diterpenes in the leaves of *C. columellaris* were pisiferal (**9**) and pisiferic acid (**10**), whereas a single diterpene was evident in *C. endlicheri* leaves, being isoozic acid (**11**), which is the isomer of the more widespread ozic acid in other species of *Callitris* [21]. In addition, sandaracopimaric acid (**1**) was identified in both species, but at lower concentrations as compared to *Widdringtonia*. The study by Simoneit et al. [21] reported 2 and 3 extensively in most other species *of Callitris*, which is similar to the pattern evident in *Widdringtonia*, but these diterpenes were not identified in the two species of *Callitris* in the current study. Nevertheless, mass spectral analysis can be used to verify if they occur in trace amounts. The identification of 12-hydroxycallitrisic acid in the resin of *C. baileyi* C.T. White by Simoneit et al. [21] is interesting since it only differs from pisiferic acid by the positioning of the carboxyl group.

The species sampled for the current study (*C. endlicheri* & *C. columellaris*) were geographically disjunct from those studied by Simoneit et al. [21]. Within Australia, *C. columellaris* is treated as three distinct species, as previously mentioned [28]. Because Simoneit et al. [21] did not identify the abietanes pisiferal (**9**) or pisiferic acid (**10**), it is likely that they sampled the actual *C. columellaris* cultivated from the coastal south-east Qld genepool, which grows in the sand dunes. The specimens of *C. columellaris* sampled for the current study were all taken from the inland temperate regions and represent the taxa that is widely recognized in Australia as *C. glaucophylla*. In the current study, we have chosen to recognize these interior specimens as a distinct chemotype.

Nevertheless, the current study constitutes the first appearance of the abietanes pisiferal (**9**) and pisiferic acid (**10**) in *Callitris*. Otherwise, they are familiar to Cupressaceae, first isolated from the etymologically related Japanese species *Chamaecyparis pisifera* (Siebold and Zucc.) Endl. [29]. The authors considered the discovery of pisiferic acid (**10**) in *C. columellaris* leaves fortuitous since it has been recognised as having significant commercial potential in the context of preservatives and topical antimicrobial therapy. It yields at 0.1% *w/w* from the leaves and is sustainably harvested, which ensures that trees are able to recover without long-term negative effects. Since 10 has a carboxylic acid moiety, it can be enriched using an organic/base partition using aqueous ammonia extraction and then neutralised with HCl to create a composition of 40–50% purity.

In the interest of confirming chemical similarity of *C. columellaris* over a wide geographic range, the specimens were sampled from several locations in Qld and NSW, which all displayed similar yields of 10. Specimens collected from as far south as Peak Hill (central NSW) to as far north as Blackall (north Qld) all yielded 10. In contrast, *C. endlicheri* was considerably variable, but a full report of the chemical character is beyond the scope of this study. Variation can be explained by the disjunct nature of its distribution since it is restricted to rocky slopes and plains, gorges, and other geographical features that are not widespread. This creates barriers to gene spread and inevitably leads to chemical divergence.

### 2.3. Structural Characterisation of Diterpenes 4–8

Most of the new structures are derivatives (4, 6–8) or an aldehyde (5) of the two known labdane isomers, *Ε*- and *Ζ*-communic acids 2 and 3, and isocommunic acid [30]. The butadiene moiety made these compounds susceptible to degradation in storage during flash chromatography using silica gel. Nevertheless, the structures were successfully assigned using 1D and 2D NMR spectra and high resolution electrospray ionization mass spectrometry (HRESIMS).

The butadiene moiety of compounds 4–6 had characteristic broad proton singlets in the olefinic region (4: δ_H_ 5.11, 5.21, 5.23 and 5.43 ppm) and were seen in the HSQC (heteronuclear single quantum coherence) spectrum to be part of two methylidenes. In the HMBC (heteronuclear multiple bond correlation) spectrum, these protons interacted with the neighbouring olefin (Table 3). The overlap of ^13^C shifts to isocommunic acid [30] showed strong agreement (Supplementary Files, Table S1) after allowing for the electronic influences of the oxidised and adjacent carbons. For example, the C12-OH shift on 4 altered most of the ^13^C shifts in the butadiene moiety, but, on 5 and 6, the shifts from C13 to C16 had <1 ppm difference as compared to isocommunic acid.

HRESIMS of 4 gave a molecular ion peak at *m/z* 357.2030 [M + Na]^+^, which is consistent with a molecular formula of C_20_H_30_O_4_. This only differs from isocommunic acid by the addition of two oxygen atoms. This molecular formula gives an index of hydrogen deficiency (IHD) of 6 with four olefinic carbons (δ_C_ 115.0, 115.5, 136.6 & 145.9 ppm) and a resonance in the acid region (δ_C_ 182.3). This indicated a tricyclic molecule. Relative to isocommunic acid, 4 does not have the C8–C17 methylidene, but instead displays two resonances consistent with an epoxide (C8 δ_C_ 60.3 ppm & C17 δ_C_ 50.4 ppm). The HSQC spectrum was used to assign the two oxirane protons at 2.82 and 2.60 ppm to C17 at δ_C_ 50.4 ppm. The HMBC spectrum indicated that these oxirane protons have a ^1^H-^13^C interaction with the quaternary C8 position, and, in the COSY spectra, a ^4^*J* ^1^H–^1^H coupling was observed between the downfield oxirane proton (δ_H_ 2.82 ppm) and one of the C7 protons (δ_H_ 1.92 ppm). Similar chemical shifts were observed in several other labdanes [31,32,33,34,35,36] with a spiro-epoxy group at C8. Another downfield resonance was observed at δ_C_ 83.8 ppm, which is consistent with an allylic alcohol, with the position established as C12 using the HMBC spectrum. A ^1^H-^13^C interaction from H16/H16’ to C12 was observed, and the reverse interaction, H12 to the olefinic resonance C16 was also seen. These observations led to the structure of 12-hydroxy-8*R*,17-spiroepoxy-isocommunic acid for 4 with the 2D NMR spectra being consistent with the proposed structure (Table 3). The configuration of C12 could not be unambiguously determined due to free rotation in the chain. In the 2D NOESY (nuclear overhauser effect spectroscopy) spectrum, a through-space interaction from the downfield C17 oxirane proton (δ_H_ 2.82 ppm) to one of the C11 protons and the C20 methyl group were seen. However, this was insufficient to assign the configuration at C12. The replacement of the C8–C17 methylidene with an oxirane also presumably affects the chemical shifts throughout the spectrum of 4 relative to isocommunic acid (Supplementary: Tables S1 and S2) [37].

Determining the configuration of spiroepoxy groups can be challenging due to the small differences in chemical shifts and the similar position for the oxiranyl hydrogens in the two isomers. Bastard et al. [32] have reported that the ^13^C shift at C17 of 8-spiroexpoxy labdanes is further downfield in the 8*R* configuration (which they refer to as α-configuration) with values ranging from δ_C_ 50–51 ppm. This contrasts with the 8*S* configuration with C17 shifts of δ_C_ 48–49 ppm. The oxirane protons are also affected with H17 shifting on the 8*R* epimer ranging from δ_H_ 2.55–2.60 ppm and δ_H_ 2.70–2.82 ppm in contrast with δ_H_ 2.20–2.30 ppm and δ_H_ 2.42–2.70 ppm on the 8*S* epimer. The difference is believed to be a consequence of equatorial vs. axial shielding effects [32]. By comparing chemicals shifts for eight labdanes with the 8*R*-configuration and five with the 8*S*-configuration (Supplementary: Tables S3–S5 and Figures S1–S3), the relationship appears to hold. While it is possible that the ^13^C NMR chemical shift of C17 on the 8*S* epimer can be shifted due to the C11–C16 moiety [36], the oxiranyl protons in 4 demonstrated shifts within the predicted range for the 8*R* configuration.

HRESIMS of 5 detected a molecular ion peak at *m/z* 341.2094 [M + Na]^+^, which is consistent with a molecular formula of C_20_H_30_O_3_ and an IHD of 6. The ^13^C NMR shifts in the butadiene moiety (C13–C16) very closely and coincided with those reported for isocommunic acid with Δδ < 0.6 and the compound differed from 4 due to the absence of the C12-OH. The ^13^C NMR spectrum of 5 lacked the oxiranyl signals and contained a C17 aldehyde with signals at δ_C_ 205.2 ppm and δ_H_ 9.56. The H17 aldehyde resonance coupled to H8 in the COSY spectrum. The configuration at C8 is also believed to be *S* as the NOESY spectrum showed that H8 interacted with H9, and there was an interaction between H17 at δ_H_ 9.56 ppm and H11 at δ_H_ 1.18 ppm, which supports the cis configuration. Thus, 5 was tentatively assigned as 8*S*-formyl-isocommunic acid and HMBC couplings corroborated this (Table 3).

Similar to 5, the ^13^C shifts on the butadiene moiety of 6 were consistent with isocommunic acid (Supplementary: Table S1). HRESIMS of 6 detected a molecular ion peak at *m/z* 319.2258 [M + H]^+^, which is consistent with a molecular formula of C_20_H_30_O_3_ and an IHD of 6. Aside from the butadiene moiety, the ^13^C and ^1^H spectrums of 6 closely resembled that of 4 (Table 3), which indicates a structure differing only from isocommunic acid by the spiroepoxy moiety. By examination of the HMBC couplings on 6 (Table 3), the structure was assigned as 8*R*,17-epoxy-isocommunic acid.

HRESIMS of 7 and 8 gave a molecular ion peak at *m/z* 341.2081 [M + Na]^+^ for both compounds, which is again consistent with a molecular formula of C_20_H_30_O_3_ and an IHD of 6. Out of the new compounds, only 7 and 8 were demonstrated to be derivatives of the communic acid cis/trans isomers [38]. The ^1^H and ^13^C spectra of both isomers (Table 4) very closely matched that of 6 (Table 3) with the exception of the butadiene chain, where the second methylidene in 6 was not observed in 7 or 8. In contrast, the olefinic carbon at C13 was substituted with a methyl group (C16), which gave a 3H singlet in the ^1^H NMR spectrum in the olefinic methyl region (**7**: δ_H_ 1.68 ppm, **8**: δ_H_ 1.77 ppm). Since the most significant differences between 7 and 8 were on the side chain moiety, it was established that they differed via isomerism at C13. A key diagnostic chemical shift at C14 can be used to distinguish between the isomers, with δ_H_ 6.30 indicating the *E*-isomer and δ_H_ 6.77 ppm for the *Z*-isomer [38]. In the current study, the H14 in **7** was observed at δ 6.33 ppm, and, on 8, H14 was observed at δ 6.76 ppm (Table 4). The ^13^C shifts of C14 are also diagnostic with δ_C_ 141.8 ppm seen for the *E*-isomer and δ_C_ 133.9 ppm for the Z-isomer [38], which were identical to the ^13^C chemical shifts for C14 on 7 and 8, respectively. Thus, **7** was assigned as 8*R*-17-epoxy-*E*-communic acid and 8 as 8*R*-17-epoxy-*Z*-communic acid.

A detailed summary of ^13^C NMR shifts for derivatives of labdane C4 acid esters are given by Barrero and Altarejos [33] and labdane C4 dimethyl derivatives by Bastard et al. [32]. However, a more comprehensive comparison to data in the current manuscript is provided in the Supplementary Files.

### 2.4. Antimicrobial and Acaricidal Activity

Regrettably, due to the low yield and instability of the spiroexpoxy communic acid derivatives (4–8), acaricidal activities were not measured for these compounds. However, the major diterpenes of *Widdringtonia* displayed very modest acaricidal activity using ticks as our model organism (Table 7, IC50 = 84–482 µg/mL). Although LC_50_ values within the range of 90–500 µg/mL are included in Table 7, only values lower than 25 µg/mL were considered interesting. In this regard, values for pisiferic acid were only moderate, but the treatments with noteworthy activity included guaiol (**12**) (6.9–15.1 µg/mL) and the sesquiterpene rich essential oil from *Widdringtonia* timber (15.5–39.8 µg/mL).

Guaiol is one of the predominating sesquiterpenes in the timber of all *Callitris* and may be considered important in the termite resistance [39], but this is the first account of activity against Acari. Since this outcome was also evident using essential oils from timbers of *Widdringtonia*, it may be feasible to correlate termite resistant timbers to resistance against Acari in general. This generates more questions about possible insect repellent activity and may translate to the ethnopharmacological context where topical applications may have alleviated ectoparasitic problems.

The antimicrobial activity of guaiol is moderate with values as low as 120–250 μg/mL against Gram-positive bacteria. The diterpenes 1 and 2 were not active at the starting concentrations used, but the spiroepoxy diterpenes were moderate to interesting, in particular 4 and 5 with MIC values ranging from 43–400 μg/mL against Gram-positive strains and 160 μg/mL against *P. aeruginosa* (Table 6). Moderate MIC values for spathulenol and guaiol were demonstrated, which ranged from 100–250 μg/mL against Gram-positives and 300 μg/mL against both Gram-negative organisms. All of these compounds are of high relative abundance in the cones of *Widdringtonia* with the yield of spathulenol at 1% by mass (Table 2). Thus, these specialised metabolites evidently provide the pharmacological basis of the traditional use of the clear, hard gum in applications consistent with antimicrobial or acaricidal activity.

The activity of 10 (pisiferic acid) was reiterated here, with results not unlike those reported previously [40], by inhibiting Gram-positive bacteria at concentrations as low as 50 µg/mL, but not Gram-negative bacteria at the concentrations tested (Table 6). In the current study, 10 was then screened against an MRSA strain and it was clear that the activity was consistently 50 µg/mL.

### 2.5. General Discussion

It is strange that both *Callitris* and *Widdringtonia* had not, until recently, had diterpenes reported, despite evident widespread occurrence and high relative abundance in leaves. This may be explained by the general trend in previous studies of the two genera to focus almost exclusively on solvent extractables from heartwood timber or volatiles from timber and leaves. In the current study, it was demonstrated that these diterpenes are not restricted to leaves, as they are also present in twigs and timber from young thin branches. Future research may demonstrate much higher yields from the cones, which has been tentatively observed in the current study.

The pronounced chemical difference between heartwood and aerial parts of the species may have ecological connotations for the genera, particularly since part of the termite and fungal resistance of the heartwood timbers. In the current study, termiticidal activities were not measured and acaricidal assays were conducted merely to address questions of a commercial interest. Nevertheless, the broad-spectrum activity of guaiol (from heartwood of *Callitris*) and widdrol/cedrol (from timbers of *Widdringtonia*) gives impetus to further investigate in this regard. Watanabe et al. [39] demonstrated that most of the essential oil components in timbers of *Callitris* repelled termites, which included components in the costol group, such as costic acid, eudesmols, collumellarin, and guaiol [39]. No similar studies have been conducted on essential oils from *Widdringtonia*.

## 3. Materials and Methods

### 3.1. Ticks Collection

Tick specimens were collected from the Indian states. Fully engorged females of *Rhipicephalus sanguineus* sensu lato, *Rhipicephalus* (*Boophilus*) *microplus*, and *Haemaphysalis bispinosa* were collected from dogs, cattle, and goats, respectively, from Assam state, North-east India. *Rhipicephalus* (*Boophilus*) *annulatus* and *Hyalomma dromedarii* fully engorged females were collected from Coimbatore, Tamil Nadu state and Ludhiana, Punjab state (South and North India, respectively). All specimens were taken back to the laboratory of Acarology, Department of Entomology, Assam Agricultural University, and then identified with the help of the taxonomical key previously described by Hoogstraal [41] and Geevarghese and Mishra [42]. Only females with healthy status and with no acaricide application history were selected to be used in the bioassay tests.

### 3.2. Adult Immersion Test

A total of 270 fully engorged females from each species (1650 individuals for all the used ticks) were washed twice with distilled water and dried on filter paper (Whatman, Kent, UK). Following the adult immersion tests (AIT) [43], preliminary concentrations of 950 and 5 µg/mL from each compound or mixture were prepared using methanol as a solvent and evaluated to determine the concentration sequence side-by-side with a control group using only methanol. Ten individuals of three replicates of each concentration were evaluated then left in large glass vials (Borosil, Mumbai, India) in a darkened incubator (Scigenics, Chennai, India). The mortality rates were observed after one week as the treatments require seven days [44]. A series of descending concentrations of 900, 800, 500, 300, 150, 50, and 10 µg/mL were then evaluated using the same method of the bioassay.

Probit analysis was then performed to determine the LC_50_, LC_99_, Slope, X^2^, and fiducial limit values using POLO-PC (LeOra, Berkeley, CA, USA) based on the mortality rates of the investigated ticks.

### 3.3. Botanical Material, Extraction, Isolation, and Nuclear Magnetic Resonance (NMR) Assignment

Leaves and thick branches were harvested from *Widdringtonia* specimens growing in Pretoria Botanic Gardens, South Africa or from private land in the Cape. Leaves from *Callitris* were harvested from remote locations in New South Wales, Australia. Voucher specimens were lodged either in the University of Johannesburg Herbarium (JRAU) or the NE Beadle Herbarium at the University of New England, Armidale, NSW, Australia.

Pulverised leaves or branches were extracted in dichloromethane. Compounds were isolated in flash chromatography over silica gel 60 (Merck) using 10%–20% ethyl acetate made up with pet ether. Compounds 1 and 2 eluted with 15% EtOAc in pet ether while all others are eluted at 20% EtOAc.

1D and 2D NMR spectra were generated on a Bruker Avance 500 MHz spectrometer using standard pulse sequences. Sandaracopimaric acid (1) spectra were matched to those provided in earlier studies of diterpenes from *Juniperus* [45,46]. The isomers of communic acid (Z- and E-) (2, 3) were matched to spectra by Fang et al. [47] and Olate et al. [38]. Spectra of the abietanes pisiferol/pisiferal (9) and pisiferic acid (10) were matched to the spectra by Pati and Mukherjee [48] and Pal et al. [49], respectively. ^1^H spectra for isoozic acid (**11**) was matched to what was provided by Martins et al. [50] and ^13^C assignments are provided here for the first time. The spectra of guaiol (12) was matched to spectra by Raharivelomanana et al. [51].

Five of the compounds are undescribed. These are structures 4–8, which were all isolated as amorphous translucent white solids: 12-hydroxy-8*R*,17-epoxy-isocommunic acid (4). HRESIMS *m/z* 357.2030 [M + Na]^+^ (calcd for C_20_H_30_NaO_4_, 357.2042, Δ 3.36 ppm); 8*S*-formyl-isocommunic acid (**5**), HRESIMS *m/z* 341.2094 [M + Na]^+^ (calcd for C_20_H_30_NaO_3_, 341.2093, Δ −0.29 ppm), 8*R*,17-epoxy-isocommunic acid (**6**), HRESIMS *m/z* 319.2258 [M + H]^+^ (calcd for C_20_H_31_O_3_, 319.2273, Δ 4.70 ppm), 8*R*-17-epoxy-*E*-communic acid (**7**), HRESIMS *m/z* 341.2081 [M + Na]^+^ (calcd for C_20_H_30_NaO_3_, 341.2093, Δ 3.52 ppm), 8*R*-17-epoxy-*Z*-communic acid (8), HRESIMS *m/z* 341.2081 [M + Na]^+^ (calcd for C_20_H_30_NaO_3_, 341.2093, Δ 3.52 ppm).

### 3.4. Determination of Minimum Inhibitory Concentrations (MIC)

The minimum inhibitory concentration (MIC) method described by Eloff [52], which is the same as that used by Clinical Laboratory Standards Institute [53], was used to determine the susceptibility of test pathogens to compounds. The organisms were *Staphylococcus epidermidis* (ATCC 12228), *Staphylococcus aureus* (ATCC 29213 & a methicillin resistant strain), *Pseudomonas aeruginosa* (ATCC 27703), *Bacillus subtilis* (University of New England strain), and *Escherichia coli* (ATCC 25922). The positive control used was tetracycline.

### 3.5. GC-MS, HRESIMS

Relative abundances of essential oil components and esterified diterpene acids were studied using gas chromatography with mass spectrometric detection (GC-MS). GC-MS analyses were performed using an Agilent Technologies 7890A GC-System coupled with an Agilent 5975C mass selective detector (triple-Axis detector, Agilent Technologies, Wilmington, DE, USA). An autosampler unit (Agilent Technologies 7693-100 positions) held samples. Separation of 1-μL injections used an HP-5MS Agilent column (30 m × 250 μm × 0.25 μm). Operating conditions were as follows: injector split ratio 25:1, temperature 250 °C, carrier gas helium, 1.0 mL/min, and constant flow. Column temperature was 50 °C (no hold) and 5 °C per minute. Then, at 280 °C, it was held at 5 min. Mass fragmentation patterns were acquired at −70 eV using a mass scan range of *m/z* 30–400.

Primary identifications were performed by comparison of mass spectra with an electronic library database [54] and confirmed using arithmetic indices, calculated relative to *n*-alkanes, when compared with values published in Adams [55] by visual comparison against mass spectral images [55]. Semi-quantification was achieved by the GC-MS operating software, using data with a minimum peak area of 0.1%, by calculating the area under the curve.

HRESIMS spectra were recorded using an AB Sciex 5600 TripleTOF mass spectrometer in positive mode.

## Figures and Tables

**Table 1:** Yields of isolates 1–12 in % (*g/g*). C.e—*Callitris endlicheri*, C.c—*C. columellaris*, W.s—*Widdringtonia schwartzii*, W.c—*W. wallichii*, W.n—*W. nodiflora*. (L)—Leaves, (Tw)—Twigs, (Ti)—Timber, (C)—cones. Sandaracopimaric acid (**1**), *Z*-communic acid (**2**), *E*-communic acid (**3**), 12-hydroxy-8*R*,17-epoxy-isocommunic acid (**4**), 8*S*-formyl-isocommunic acid (**5**), 8*R*,17-epoxy-isocommunic acid (**6**), 8*R*-17-epoxy-*E*-communic acid (**7**), 8*R*-17-epoxy-*Z*-communic acid (**8**), pisiferal (**9**), pisiferic acid (**10**), isoozic acid (**11**), and guaiol (**12**).

-	1	2	3	4	5	6	7	8	9	10	11	12
*C.e* (L)	2 × 10^−4^	-	-	-	-	-	-	-	-	-	0.06	0.1
*C.c* (L)	0.05	-	-	-	-	0.01	-	-	0.04	0.20	-	0.1
*W.s* (L)	0.10	0.01	-	-	4 × 10^−3^	2 × 10^−3^	3 × 10^−3^	-	-	-	-	-
*W.s* (Tw)	0.04	2 × 10^−3^	-	-	-	4 × 10^−3^	-	-	-	-	-	-
*W.s* (Ti)	0.15	-	-	-	-	5 × 10^−3^	-	-	-	-	-	-
*W.w* (L)	0.10	0.01	0.01	7 × 10^−4^	0.01	0.01	0.01	3 × 10^−5^	-	-	-	-
*W.w* (Tw)	0.07	-	-	-	-	4 × 10^−3^	-	-	-	-	-	-
*W.w* (Ti)	0.12	-	-	4 × 10^−5^	-	0.01	-	4 × 10^−4^	-	-	-	-
*W.n* (L)	0.23	0.04	0.03	2 × 10^−3^	0.01	0.02	0.01	1 × 10^−3^	-	-	-	-
*W.n* (Tw)	0.02	0.01	-	-	-	4 × 10^−4^	-	-	-	-	-	-
*W.n* (Ti)	0.24	0.02	-	-	8 × 10^−3^	0.01	0.01	-	-	-	-	-
*W.n* (C)	0.05	0.37	0.18	-	-	0.42	0.15	0.10	-	-	-	-

**Table 2:** Yields of specific terpenes and pisiferol across the species and plant parts in percent (*g/g*). C.e = *Callitris endlicheri*, C.c = *C. columellaris*, W.s = *Widdringtonia schwartzii*, W.w = *W. wallichii*, W.n = *W. nodiflora*. (L) = Leaves, (Tw) = Twigs, (Ti) = Timber, (C) = cones: Pi-OH = pisiferol, Car-Ox = caryophyllene oxide, F-Ac = fenchyl acetate, B-Ac = bornyl acetate, Th-ene = thujopsene, Spath = spathulenol, Hin+H = hinokiic acid, Cup+H = cuparenic acid.

-	Pi-OH	Car-Ox	F-Ac	B-Ac	Th-ene	Spath	Hin + H	Cup + H
*C.e* (leaf)	-	-	-	-	-	-	-	-
*C.c* (leaf)	4 × 10^−4^	0.04	0.14	0.27	-	-	-	-
*W.s* (leaf)	-	-	-	-	-	-	-	-
*W.s* (twig)	-	-	-	-	0.03	-	0.03	-
*W.s* (wood)	-	-	-	-	0.29	0.07	0.24	0.01
*W.w* (leaf)	-	-	-	-	3 × 10^−4^	0.01	-	-
*W.w* (twig)	-	-	-	-	-	-	-	-
*W.w* (wood)	-	-	-	-	-	-	-	-
*W.n* (leaf)	-	-	-	-	0.01	1.2 × 10^−7^	-	-
*W.n* (twig)	-	-	-	-	-	0.04	-	-
*W.n* (wood)	-	-	-	-	-	-	-	-
*W.n* (cone)	-	-	-	-	0.13	1.28	-	-

**Table 3:** ^13^C and ^1^H NMR data for new compounds. 12-hydroxy-8*R*,17-epoxy-isocommunic acid (**4**), 8*S*-formyl-isocommunic acid (**5**), and 8*R*,17-epoxy-isocommunic acid (**6**). HMBC, heteronuclear multiple bond correlation.

	4			5			6		
-	^13^C	^1^H	HMBC	^13^C	^1^H	HMBC	^13^C	^1^H	HMBC
**1**	38.5	1.65, dt (2.4, 12.98)	17	38.6	1.84, m	10, 20	39.2	1.81, m	-
		1.02–1.09, m	-	-	1.03, m	-	-	1.07, td (4.04, 13.86)	-
**2**	19.4	1.79, qt (3.6, 13.9)	1, 3	19.2	1.85, m	3, 20	19.4	1.82, m	3
		1.49, dt (3.3, 13.9)	-	-	1.5, m	-	-	1.51, dt (13.9, 2.86)	-
**3**	37.9	2.17, brd (13.7)	18	38	2.19, m	5, 18	37.9	2.19, brd (13.15)	2, 18
		1.07–1.14, m	-	-	1.07, m	-	-	1.05, d (13.15)	-
**4**	44.1	-	3, 5, 18	43.9	-	2, 3, 5, 18	44.2	-	3, 18
**5**	55.6	1.27 m	3, 6, 7, 17, 18	55.6	1.19, dd (2.58, 12.39)	1, 3, 18, 20	55.9	1.29	1, 3, 6, 7, 17, 18
**6**	23.5	2.03–2.1 m, 2H	5	21.7	1.97, d (3.86)	5, 7	23.5	2.03, m	5, 16
				-	1.86, m	-	-	1.64, q (7.94)	-
**7**	36.9	1.92, tdd (1.97, 5.6, 12.2)	5, 6, 11, 20	27.4	1.78, m	8, 9	37.1	1.83, m	20
-	-	1.44, dt (3.4, 12.2)	-	-	1.36, dd (4.3, 12.6)	-	-	1.41, dt (3.41, 12.27)	-
**8**	60.3	-	6, 7, 9, 11, 20	54.1	2.32, m	7, 11, 17	59.1	-	6, 7, 9, 11, 20
**9**	47.9	1.88, d (7.1)	7, 11, 12, 17	50.3	1.22, m	1, 7, 8, 11, 12, 20	53.4	1.48, dd (2.45, 6.72)	1, 7, 11, 12, 17
**10**	40.6	-	6, 9, 11	38.5	-	1, 5, 11, 20	41	-	6, 9, 11, 17
**11**	25.5	1.08–1.15, m	7, 9, 12	28.4	1.64, m	9, 12	21.7	1.28, m	9, 12, 16
-	-	1.29–1.36, m	-	-	1.18, m	-	-	0.92, m	-
**12**	83.8	4.85, dd (3.2, 7.7)	9, 11, 14, 16	32.7	2.21, m	11, 14, 16	33.7	2.36, ddd (5.01, 11.06, 14.16)	9, 11, 14, 16
-	-	-	-	-	2.06, m	-	-	2.06, m	-
**13**	145.9	-	11, 12, 14, 15, 16	146.4	-	11, 12, 14, 15	147.2	-	11, 12, 14, 15, 16
**14**	136.6	6.31, dd (11.36, 17.5)	15, 16	138.6	6.3, dd (10.95, 17.67)	12, 15, 16	138.8	6.32, dd (10.86, 17.62)	12, 15, 16
**15**	115	5.11, d (11.36)	14, 16	113.7	5.15, d (17.67)	-	113.7	5.05, br dd (0.89, 10.86)	16
-	-	5.43, d (17.5)	-	-	5.05, d (10.95)	-	-	5.27, d (17.62)	-
**16**	115.5	5.21, br s	12, 14, 15	116.4	4.98, s	12, 14	116	4.97, s, 2H	12, 14, 15
-	-	5.23, br s	-	-	4.93, s	-	-	-	-
**17**	50.4	2.60, brd (3.89)	7, 9	205.2	9.56, d (4.6)	7, 8	50.6	2.53, d (4.34)	7
	-	2.82, dd (1.97, 3.89)	-	-	-	-	-	2.76, dd (1.87, 4.34)	-
**18**	29.1	1.27, s, 3H	3, 5	29	1.26, s, 3H	5, 13	29.1	1.28, s, 3H	3, 5
**19**	182.3	-	3, 5, 18	182.7	-	3, 5	183.6	-	3, 18
**20**	13.3	0.67, s, 3H	9, 11	12.7	0.75, s, 3H	1, 9, 11	13.2	0.71, s, 3H	5, 9

**Table 4:** ^13^C and ^1^H NMR spectral data for tentatively assigned compounds. 8*R*-17-epoxy-*E*-communic acid (7), 8*R*-17-epoxy-*Z*-communic acid (8).

	*E*- (7)		*Z*- (8)	
	^13^C	^1^H	^13^C	^1^H
1	39.7	1.1, n.d.	39.8	1.1, n.d.
		1.75, n.d.		1.75, n.d.
2	19.4	1.5, n.d.	19. 5	1.5, n.d.
		1.8, n.d.		1.8, n.d.
3	37.9	1.04, n.d.	37.9	1.04, n.d.
		2.17, n.d.		2.17, n.d.
4	44.2	-	44.2	-
5	55.8	1.29, ^1^H	55.8	1.29, ^1^H
6	23.4	1.7, n.d.	23.4	1.7, n.d.
		2.1, n.d.		2.1, n.d.
7	36.7	1.41, n.d.	36.7	1.41, n.d.
		1.85, n.d.		1.85, n.d.
8	58.8	-	58.9	-
9	54.3	1.6, ^1^H, n.d.	54.5	1.6, ^1^H, n.d.
10	41	-	41.1	-
11	21.1	1.99, n.d.	20.2	1.99, n.d.
		1.72, n.d.		1.72, n.d.
12	135.1	5.5, brt (6.9)	132.8	5.4, brt (7.6)
		-		-
13	132.7	-	130.9	-
14	141.8	6.33, dd (10.8, 17.2)	133.9	6.76, ddd (0.7, 10.9, 17.5)
15	110.2	5.04, d (17.2)	113.4	5.16, dd (0.8, 17.5)
		4.89, d (10.8)		5.07, dt (0.8, 10.9)
16	23.6	1.68, 3H s	19.9	1.77, 3H brs
		-		-
17	50.3	2.56, d (4.3)	50.3	2.55, d (4.3)
		2.7, dd (1.8, 4.3)		2.7, dd (1.8, 4.3)
18	29.2	1.27, 3H s	29.2	1.27, 3H s
19	183.5	-	183.5	-
20	13.3	0.78, 3Hs	13.3	0.77, 3Hs

**Table 5:** Essential oil components from plant parts of *Widdringtonia*. W.s—*Widdringtonia schwartzii*, W.n—*W. nodiflora*. (L)—Leaves, (Tw)—Twigs, (Ti)—Timber.

	AI	Pub. AI	W.n (L)	W.n (Tw)	W.n (Ti)	W.s (L)	W.s (Ti)
α-Thujene	920	924	3.1	0.6	0.3	40.7	-
α-Pinene	927	932	62.1	37.0	4.5	20.5	-
Camphene	943	946	0.4	0.2	-	-	-
Thuja-2,4(10)-diene	946	953	0.4	0.5	-	-	-
Sabinene	965	969	5.7	0.8	-	-	-
β-Pinene	971	974	4.1	2.7	0.3	-	-
Myrcene	980	988	3.6	4.4	-	-	-
α-Phellandrene	1001	1002	0.2	0.2	-	-	-
ƍ-3-Carene	1010	1008	1.2	0.3	-	-	-
α-Terpinene	1018	1014	1.2	2.1	-	-	-
Limonene	1023	1024	-	0.7	-	-	-
β-Phellandrene	1024	1025	1.5	0.7	-	-	-
ƴ-Terpinene	1051	1054	2.7	0.7	-	-	-
Terpinolene	1080	1086	0.9	0.4	-	-	-
α-Campholenal	1123	1122	0.4	0.8	-	-	-
E-pinocarveol	1138	1135	0.2	0.5	-	-	-
Camphor	1144	1141	0.1	0.3	-	-	-
p-Mentha-1,5-dien-8-ol	1171	1166	0.4	1.0	-	-	-
Terpinen-4-ol	1178	1174	2.9	3.0	-	-	-
α-Terpineol	1196	1186	0.6	1.6	-	-	-
Verbenone	1208	1204	-	1.0	-	-	-
E-Caryophyllene	1419	1417	0.3	1.6	0.3	1.7	-
Z-Thujopsene	1435	1429	0.2	2.2	15.4	0.8	11.8
Aromadendrene	1438	1439	-	0.6	0.4	-	-
α-Caryophyllene	1455	1452	1.1	5.1	4.3	6.9	-
Z-Muurola-4(11),5-diene	1462	1465	-	0.5	-	-	-
ƴ-Muurolene	1475	1478	0.2	1.8	2.2	1.9	-
Germacrene D	1481	1480	0.8	6.1	3.6	4.5	18.3
β-Selinene	1488	1489	-	0.1	-	-	-
Bicyclogermacrene	1495	1500	0.8	3.9	2.5	6.1	-
α-Muurolene	1498	1500	0.2	0.6	1.5	-	-
α-Cuparene	1508	1504	-	-	3.0	-	14.3
ƴ-Cadinene	1513	1513	0.2	1.3	1.7	1.4	-
ƍ-Cadinene	1518	1522	0.5	2.8	4.1	3.7	-
Spathulenol	1578	1577	0.9	2.0	4.2	3.5	2.9
Globulol	1587	1590	0.2	0.3	-	-	-
Widdrol	1597	1599	-	0.1	4.3	-	3.2
Cedrol	1609	1600	-	0.5	10.4	-	10.1
Epi-α-cadinol	1635	1638	-	0.3	1.3	-	5.9
α-Muurolol	1644	1644	-	-	-	2.2	13.5
Cubenol	1645	1645	-	-	-	-	5.3
α-Cadinol	1658	1652	0.5	1.1	7.2	-	-
Widdrenal	1708	1708	-	-	1.8	-	11.0

**Table 6:** Minimum inhibition concentration (MIC) values (μg/mL) for compounds 1–2, 4–8, and 10. Sandaracopimaric acid (1), *Z*-communic acid (2), 12-hydroxy-8*R*,17-epoxy-isocommunic acid (4), 8*S*-formyl-isocommunic acid (5), 8*R*,17-epoxy-isocommunic acid (6), 8*R*-17-epoxy-*E*-communic acid (7), 8*R*-17-epoxy-*Z*-communic acid (8), and pisiferic acid (10). The organisms were *Staphylococcus epidermidis* (ATCC 12228), *Staphylococcus aureus* (ATCC 29213), *Pseudomonas aeruginosa* (ATCC 27703), *Bacillus subtilis* (University of New England strain), and *Escherichia coli* (ATCC 25922). Spath = spathulenol encapsulated using equimolar concentration of α-cyclodextrin. * Also tested against a methicillin resistant Staphylococcus aureus (MRSA) strain, which gave the same MIC value. Tet = positive control tetracycline.

	1	2	4	5	6	7	8	10	12	Spath	Tet
*S. aureus*	>1500	>1500	160	170	1500	400	400	50 *	250	160	0.13–0.25
*S. epi*	>1500	>1500	160	76	1500	400	400	50	170	100	0.5–0.75
*B. sub*	>1500	>1500	160	43	375	178	178	50	120	100	0.13–0.15
*P. aeru*	>1500	>1500	160	170	>1500	>1500	>1500	>200	300	>200	0.75–1.0
*E. coli*	>1500	>1500	>160	>170	>1500	>1500	>1500	>200	300	>200	0.25–0.75

**Table 7:** Acaricidal activity against tick species for compounds. Sandaracopimaric acid (1), *Z*-communic acid (2), pisiferal (9), pisiferic acid (10), guaiol (12), and essential oil from *W. nodiflora* timber (EO-W.n). LC_99_ values were extrapolated using probit analysis.

	µg/mL	1	2	9	10	12	EO-W.n
*Haemaphysalis bispinosa*	LC_50_	105.5	84.8	194.3	34.9	6.9	15.5
	LC_99_	3255.4	1444.6	41,850.4	13,129.7	6697.1	8335.1
*Hyalomma dromedarii*	LC_50_	450.8	482.9	359.8	230.8	15.1	28.6
	LC_99_	9716.7	6970.4	16,334.5	18,154.8	6641.5	56,104.1
*Rhipicephalus (Boophilus) annulatus*	LC_50_	390.7	216.1	320.1	255.1	12.3	39.8
	LC_99_	5584.4	9445.1	52,752.1	54,206.2	23,301.8	122,740.1
*Rhipicephalus (Boophilus) microplus*	LC_50_	365.2	201.1	420.9	220.4	11.4	28.4
	LC_99_	7359.8	8472.1	203,209.3	91,284.2	18,684.4	129,759.7
*Rhipicephalus sanguineus* sensu lato	LC_50_	166.7	189.8	255.7	88.2	9.1	22.5
	LC_99_	6952.2	3051.8	50,268.5	15,145.4	14,856.2	84,962.1

## Supplementary Materials

The following are available online at https://www.mdpi.com/2079-6382/9/4/173/s1. Figure S1: Standard chemical shifts for 8-spiroepoxy labdanes in the *R* and *S* configurations, as proposed by Bastard et al. [32]. Figure S2: Labdanes with 8*R*-spiroepoxy conformation. Figure S3: Labdanes with 8*S*-spiroepoxy conformation. Table S1: Comparison of ^13^C shifts (ppm) from isocommunic acid with ^13^C shifts from compounds 4–6 by subtraction. Columns 4–6 show a difference in ^13^C shifts compared to ^13^C shifts of isocommunic acid. Table S2: Comparison of ^13^C shifts for **7** and **8** to cis (Ζ-) and trans (Ε-) communic acids. Column 1 gives ppm difference of **7** and **8** ^13^C shifts compared to Ε-communic acid and column 2 compares to Ζ-communic acid. Table S3: ^13^C NMR shifts (δ, CDCl3) for 8*R*-spiroexpoxy labdanes taken from the literature compared to 4 and 6 and the aldehyde **5**: AR [31]; BR [32]; CR and DR [33]; ER, FR and GR [35]; HR [34]. See Figure S2 for the structures, Table S4: ^13^C NMR shifts (δ, CDCl3) for 8*S*-spiroexpoxy labdanes taken from the literature: AS [32]; BS and CS [34]: DS and ES [36]. See Figure S3 for the structures. Table S5: ^1^H NMR of vinylic protons at the C17 methylene position in the 8*R* or 8*S* spiroepoxy moiety: AR [31]; BS and CS [35]; DS and ES [36].

## References

[1] Farjon A., February E., Higgins S., Fox S., Raimondo D. (2013). Widdringtonia cedarbergensis. Red List.

[2] Smith C.A. (1955). Early 19th century records of the Clanwilliam cedar (*Widdringtonia juniperoides* Endl.). J. S. Afr. For. Assoc..

[3] Van Wyk V., Van Wyk P., Van Wyk B.-E. (2008). Photo Guide to Trees of Southern Africa.

[4] Manders P.T., Botha S.A., Bond W.J., Meadows M.E. (1990). The enigmatic Clanwilliam cedar. Veld Flora.

[5] Erdtman H., Thomas B.R. (1958). The chemistry of the natural order Cupressales. 20. Heartwood constituents of the genus *Widdringtonia*. Acta Chem. Scand..

[6] Farjon A. (2013). Widdringtonia schwarzii. Red List.

[7] Farjon A. (1998). World Checklist and Bibliography of Conifers.

[8] Enzell C., Erdtman H. (1958). The chemistry of the natural order cupressales -XXI.; Cuparene and Cuparenic acid, two sesquiterpenic compounds with a new carbon skeleton. Tetrahedron.

[9] Enzell C. (1962). The chemistry of the natural order cupressales 47: The structures and absolute configurations of Widdrol and Widdrol-alpha-epoxide. Acta Chem. Scand..

[10] Kamatou G.P.P., Viljoen A.M., Özek T., Başer H.C.K. (2010). Chemical composition of the wood and leaf oils from the “Clanwilliam Cedar” (*Widdringtonia cedarbergensis* J.A. Marsh): A critically endangered species. S. Afr. J. Bot..

[11] Green C.L., Wood A.B., Robinson J.M. (1988). A re-examination of Mulanje cedarwood oil (*Widdringtonia whytei* Rendle). Flavour Fragr. J..

[12] Norin T. (1961). The chemistry of the natural order cupressales: 40. The structure of thujopsene and hinokiic acid. Acta Chem. Scand..

[13] Tunalier Z., Kirimer N., Baser K.H.C. (2004). A potential new source of cedarwood oil: *Juniperus foetidissima* Willd. J. Essent. Oil Res..

[14] Gadek P.A., Alpers D.L., Heslewood M.M., Quinn C.J. (2000). Relationships within Cupressaceae sensu lato: A combined morphological and molecular approach. Am. J. Bot..

[15] Farjon A. (2005). A Monograph of Cupressaceae and Sciadopitys.

[16] Sadgrove N., Jones G.L. (2014). Medicinal compounds, chemically and biologically characterised from extracts of Australian *Callitris endlicheri* and *C. glaucophylla* (Cupressaceae): Used traditionally in Aboriginal and colonial pharmacopoeia. J. Ethnopharmacol..

[17] Oprava A., Leach D.N., Beattie K., Connellan P., Forster P.I., Leach G., Buchbauer G., Shepherd K., Deseo M. (2010). Chemical composition and biological activity of the essential oils from native Australian *Callitris* species. Planta Med..

[18] Doimo L. (2001). Azulenes, Costols and γ-lactones from cypress-pines (*Callitris columellaris*, *C. glaucophylla* and *C. intratropica*) distilled oils and methanol extracts. J. Essent. Oil Res..

[19] Brecknell D.J., Carman R.M. (1979). Novel sesquiterpene lactones from *Callitris columellaris* heartwood. Aust. J. Chem..

[20] Doimo L., Fletcher R., D′Arcy B.R. (1999). Comparison of the γ-lactone content of oils and extracts from White Cypress Pine (*Callitris glaucophylla* Thompson and Johnson). J. Essent. Oil Res..

[21] Simoneit B.R.T., Cox R.E., Oros D.R., Otto A. (2018). Terpenoid compositions of resisn from *Callitris* species (Cupressaceae). Molecules.

[22] Kennedy M.J., Astridge D., De Faveri S., Fay H., Firrell M., Grice K., Halfpapp K., Hargreaves J.R., Lyndal-Murphy M., Nolan B. (2008). Final Report: Commercial Products from Bio-active Extractives in Cypress Milling Residues: FWPA Project PN04.2006.

[23] Coates Palgrave K. (1977). Trees of Southern Africa.

[24] Sugimoto N., Kuroyanagi M., Kato T., Sato K., Tada A., Yamazaki T., Tanamoto K. (2005). Identification of the main constituents in sandarac resin, a natural gum base. J. Food Hyg. Soc. Jpn..

[25] Carpenter C.D., O′Neill T., Picot N., Johnson J.A., Robichaud G.A., Webster D., Gray C.A. (2012). Anti-mycobacterial natural products from the Canadian medicinal plant *Juniperus communis*. J. Ethnopharmacol..

[26] Fernandes F.H., Guterres Z.d.R., Violante I.M.P., Lopes T.F.S., Garcez W.S., Garcez F.R. (2015). Evaluation of mutagenic and antimicrobial properties of brown propolis essential oil from the Brazilian Cerrado biome. Toxicol. Rep..

[27] Brophy J.J., Goldsack R.J., Forster P.I., Copeland L.M., O’Sullivan W., Rosefelds A.C. (2007). Chemistry of the Australian Gymnosperms. Part IX. The leaf oils of the Australian members of the genus *Callitris* (Cupressaceae). J. Essent. Oil Res..

[28] Thompson J., Johnson L.A.S. (1986). *Callitris Glaucophylla*, Australia’s ‘White Cypress Pine’—A new name for an old species. Telopea.

[29] Yatagai M., Takahashi T. (1980). New diterpenes from *Chamaecyparis pisifera*. Phytochemistry.

[30] Soliman A.F., Naeem Z.M., Khalil A.T., Shimizu K., El-Sharkawy S.H. (2018). Microbial transformation of the labdane diterpene 13-epi-cupressic acid. World J. Pharm. Sci..

[31] Fang J.-M., Sou Y.-C., Chiu Y.-H., Cheng Y.-S. (1993). Diterpenes from the bark of *Juniperus chinensis*. Phytochemistry.

[32] Bastard J., Duc D.K., Fetizon M., Francis M.J., Grant P.K., Weavers R.T., Kaneko C. (1984). CMR spectroscopy of labdanic diterpenes and related substances. J. Nat. Prod..

[33] Barrero A.F., Altarejos J. (1993). ^13^C NMR data for labdane diterpenes. Magn. Reson. Chem..

[34] Morita H., Itokawa H. (1987). Cytotoxic and antifungal diterpenes from the seeds of *Alpinia galanga*. Planta Med..

[35] Schmeda-Hirschmann G., Astudillo L., Sepulveda B., Rodriguez J.A., Theoduloz C., Yanez T., Palenzuela J.A. (2005). Gastroprotective effect and cytotoxicity of natural and semisynthetic labdane diterpenes from *Araucaria araucana* resin. Zeitzchrift Fur Nat. CJ. Biosci..

[36] Sob S.V.T., Tane P., Ngadjui B.T., Connoly J.D., Ma D. (2007). Trypanocidal labdane diterpenoids from teh seeds of *Aframomum aulacocarpos* (Zingiberaceae). Tetrahedron.

[37] Martin N.H., Brown J.D. (2000). A new graphical model for proton NMR (de)shielding over a carbon-carbon double bond to replace the shielding cone model. Int. J. Mol. Sci..

[38] Olate V.R., Usandizaga O.G., Schmeda-Hirschmann G. (2011). Resin diterpenes from *Austrocedrus chilensis*. Molecules.

[39] Watanabe Y., Mihara R., Mitsunaga T., Yoshimura T. (2005). Termite repellent sesquiterpenoids from *Callitris glaucophylla* heartwood. J. Wood Sci..

[40] Yatagai M., Nakatani N. (1994). Antimite, antifly, antioxidative, and antibacterial activities of pisiferic acid and its congeners. J. Jpn. Wood Res. Soc..

[41] Hoogstraal H. (1956). African Ixodoidea Vol. 1: Ticks of the Sudan.

[42] Geevarghese G., Mishra A.C. (2011). Haemaphysalis Ticks of India.

[43] Drummond R.O., Ernst S.E., Trevino J.L., Gladney W.J., Graham O.H. (1973). *Boophilus annulatus* and *Boophilus microplus*: Laboratory test of insecticides. J. Econ. Entomol..

[44] Oliveira P.R., Bechara G.H., Camargo-Mathias M.I. (2008). Evaluation of the cytotoxic effects of fipronil on ovaries of semi-engorged *Rhipicephalus sanguineus* (Latreille, 1806)(Acari: Ixodidae) tick female. Food Chem. Toxicol..

[45] Muto N., Tomokuni T., Haramoto M., Tatemoto H., Nakanishi T., Inatomi Y., Murata H., Inada A. (2008). Isolation of apoptosis- and differentiation-inducing substrances toward human promyelocytic leukemia HL-60 cells from leaves of *Juniperus taxifolia*. Biosci. Biotechnol. Biochem..

[46] Sakar M.K., Er N., Ercil D., Olmo D.E., Feliciano A.A. (2002). (-)-Desoxypodophyllotoxin and diterpenoids from *Juniperus nana* Willd. berries. Acta Pharm. Turc..

[47] Fang J.-M., Chen Y.-C., Wang B.-W., Cheng Y.-S. (1996). Terpenes from the heartwood of *Juniperus chinensis*. Phytochemistry.

[48] Pati L.C., Mukherjee D. (2004). Stereocontrolled total synthesis of (±)-pisiferol and (±)-pisiferal. Tetrahedron Lett..

[49] Pal S.K., Gupta P.D., Mukherjee D. (2002). Stereocontrolled total syntheses of (±)-pisiferic acid and (±)-O-methylpisiferic acid. Tetrahedron.

[50] Martins D., Hamerski L., Alvarenga S.A.V., Roque N.F. (1999). Labdane dimers from *Xylopia aromatica*. Phytochemistry.

[51] Raharivelomanana P., Bianchini J.-P., Cambon A., Azzaro M., Faure R. (1995). Two-dimensional NMR of sesquiterpenes. 8-complete assignment of ^1^H and ^13^C NMR spectra of seven sesquiterpene alcohols from *Neocallitropsis pancheri*. Magn. Reson. Chem..

[52] Eloff J.N. (1998). A sensitive and quick microplate method to determine the minimal inhibitory concentration of plant extracts for bacteria. Planta Med..

[53] CLSI (2017). Performance standards for antimicrobial susceptibility testing; 23rd informational supplement. CLSI document M100-S27.

[54] NIST NIST Chemistry WebBook -NIST Standard Reference Database Number 69. http://webbook.nist.gov/chemistry/.

[55] Adams R.P. (2007). Identification of Essential oil Components by Gas Chromatography/Mass Spectrometry.

