# Supplementary material for: New Labdanes with Antimicrobial and Acaricidal Activity: Terpenes of Callitris and Widdringtonia (Cupressaceae)
Source: Antibiotics (Basel). 2020 Apr 11;9(4):173. doi: 10.3390/antibiotics9040173 (PMC7235842; doi:10.3390/antibiotics9040173)
Supplement: Supplementary file 1 [file antibiotics-09-00173-s001.pdf]

antibiotics

MDPI

Supplementary files

Table S1. Comparison of 13C shifts (ppm) from isocommunic acid with 13C shifts from compounds 4–6 by subtraction. Columns 4–6 show difference in 13C shifts compared to 13C shifts of isocommunic acid.

Heading	Δ ^{13}C	δ ^{13}C–δ ^{13}C		
**No.**	**Isocommunic Acid**	**4**	**5**	**6**
1	39.9	1.4	1.3	0.7
2	19.8	0.4	0.6	0.4
3	37.9	0.0	−0.1	0.0
4	44.1	0.0	0.2	−0.1
5	56.1	0.5	0.5	0.2
6	22.1	−1.4	0.4	−1.4
7	38.7	1.8	11.3	1.6
8	147.9	87.6	93.8	88.8
9	56.6	8.7	6.3	3.2
10	40.4	−0.2	1.9	−0.6
11	26.0	0.5	−2.4	4.3
12	30.2	−53.6	−2.5	−3.5
13	147	1.1	0.6	−0.2
14	139	2.4	0.4	0.2
15	113.2	−1.8	−0.5	−0.5
16	115.5	0.0	−0.9	−0.5
17	106.4	56.0	−98.8	55.8
18	28.9	−0.2	−0.1	−0.2
19	183.6	1.3	0.9	0.0
20	12.8	−0.5	0.1	−0.4

Table S2. Comparison of ^{13}C shifts for 7 and 8 to cis (*Z*-) and trans (*E*-) communic acids. Column 1 gives ppm difference of 7 and 8 ^{13}C shifts compared to *E*-communic acid and column 2 compares to *Z*-communic acid.

Heading	Column 1			Column 2		
	δ ^{13}C	δ ^{13}C–δ ^{13}C		δ ^{13}C	δ ^{13}C–δ ^{13}C	
No.	*E*-comm	**7** (E-)	**8** (Z-)	*Z*-comm	**7** (E-)	**8** (Z-)
1	39.5	-0.2	-0.3	39.5	-0.2	-0.3
2	20.1	0.7	0.7	20.1	0.7	0.7
3	38.1	0.2	0.2	38.1	0.2	0.2
4	44.4	0.2	0.2	44.4	0.2	0.2
5	56.5	0.7	0.7	56.5	0.7	0.7
6	26.0	2.6	2.6	26.0	2.6	2.6
7	38.7	2.0	2.0	38.7	2.0	2.0
8	148.1	89.3	89.3	148.1	89.3	89.3
9	56.6	2.3	2.1	56.9	2.6	2.4
10	40.6	-0.4	-0.5	40.6	-0.4	-0.5
11	23.5	2.4	3.3	23.5	2.4	3.3
12	134.1	-1.0	1.3	131.9	-3.2	-0.9
13	133.6	0.9	2.7	131.7	-1.0	0.8
14	141.8	0.0	7.9	133.9	-7.9	0.0
15	110.1	-0.1	-3.3	113.4	3.2	0.0
16	12.0	-11.6	-7.9	12.0	-11.6	-7.9
17	107.9	57.6	57.6	108.0	57.7	57.7
18	29.2	0.0	0.0	29.2	0.0	0.0

19	184.4	1.0	0.9	184.4	1.0	0.9
20	13.0	-0.3	-0.3	13.0	-0.3	-0.3

Table S3. 13C NMR shifts (ẟ, CDCl3) for 8*R*-spiroexpoxy labdanes taken from the literature compared to 4 and 6 and the aldehyde 5: AR [1]; BR [2]; CR & DR [3]; ER, FR & GR [4]; HR [5]. See Figure S2 for the structures.

Epimer	8R	8R	8R	8R	8R	8R	8R	8R	8R	8R	8R	8R	8R
**Figure**	**AR**	**BR**	**CR**	**DR**	**ER**	**FR**	**GR**	**HR**	**4**	**5**	**6**	**7**	**8**
1	40.6	38.9	40.56	42.23	39.4	39.3	39.3	39.2	38.5	38.6	39.2	39.7	39.8
2	19.8	18.6	19.21	19.27	19.3	18.2	19.2	18.7	19.4	19.2	19.4	19.4	19. 5
3	37.7	41.8	37.99	37.84	37.8	35.3	37.7	41.8	37.9	38.0	37.9	37.9	37.9
4	43.9	33.3	44.01	43.89	44.0	40.2	43.9	33.4	44.1	43.9	44.2	44.2	44.2
5	54.8	54.9	55.22	55.08	55.7	55.7	55.7	54.5	55.6	55.6	55.9	55.8	55.8
6	22.9	24.8	23.15	23.26	23.0	21.8	23.3	21.7	23.5	21.7	23.5	23.4	23.4
7	36.0	36.6	36.07	36.21	39.1	38.9	39.1	39.7	36.9	27.4	37.1	36.7	36.7
8	58.1	59.3	58.24	58.93	59.0	58.9	58.8	58.9	60.3	54.1	59.1	58.8	58.9
9	57.4	53.1	57.45	60.49	53.9	51.6	53.8	55.1	47.9	50.3	53.4	54.3	54.5
10	40.8	40.2	39.47	40.4	41.0	38.6	40.9	40.2	40.6	38.5	41	41	41.1
11	140.3	16.1	123.09	65.15	19.5	19.3	19.3	22.5	25.5	28.4	21.7	21.1	20.2
12	135.8	45.0	138.65	66.06	36.8	36.7	36.7	160.4	83.8	32.7	33.7	135.1	132.8
13	197.0	208.9	71.19	51.99	30.0	30.0	30.2	133.8	145.9	146.4	147.2	132.7	130.9
14	27.9	29.8	76.88	-	39.6	39.7	35.3	36.1	136.6	138.6	138.8	141.8	133.9
15	-	-	14.7	-	60.9	60.8	63.0	197.6	115.0	113.7	113.7	110.2	113.4
16	-	-	25.4	20.3	19.6	19.6	19.5	193.7	115.5	116.4	116	23.6	19.9
17	50.5	50.7	50.33	50.63	50.4	50.6	50.2	50.5	50.4	205.2	50.6	50.3	50.3
18	28.8	21.5	28.77	28.75	29.0	27.0	28.9	33.5	29.1	29.0	29.1	29.2	29.2
19	180.0	33.4	177.52	177.3	183.2	67.9	183.3	21.7	182.3	182.7	183.6	183.5	183.5
20	13.9	14.5	13.59	14.42	12.9	15.2	12.9	14.7	13.3	12.7	13.2	13.3	13.3

Table S4. 13C NMR shifts (ẟ, CDCl3) for 8*S*-spiroexpoxy labdanes taken from the literature: AS [2]; BS & CS [5]: DS & ES [6]. See Figure S3 for the structures.

Epimer	8S	8S	8S	8S	8S
**Figure**	**AS**	**BS**	**CS**	**DS**	**ES**
1	38.8	39.6	39.3	40.7	39.6
2	18.5	18.4	18.6	21.6	19.0
3	42.0	42.0	41.9	43.6	42.3
4	33.4	33.6	33.5	34.9	33.9
5	55.0	52.8	52.4	54.9	53.7
6	20.1	20.0	20.0	20.1	20.5
7	35.9	39.6	35.8	37.5	36.3
8	57.1	57.6	57.5	59.7	57.7
9	52.0	55.2	55.0	56.6	55.4
10	40.0	40.0	39.6	41.3	40.2
11	15.6	22.4	22.8	21.2	20.6
12	45.4	161.2	142.8	133.7	140.1
13	208.1	136.0	124.8	139.2	129.1
14	29.7	36.0	25.4	72.3	70.5
15	-	198.0	65.3	66.5	64.5
16	-	194.0	171.2	64.3	65.4
17	48.7	48.8	49.0	50.7	49.4
18	21.7	33.6	33.5	34.5	33.9
19	33.4	21.6	21.7	22.6	22.1
20	14.4	14.8	14.6	15.7	15.0

Table S5. 1H NMR of vinylic protons at the C17 methylene position in the 8*R* or 8*S* spiroepoxy moiety: AR [1]; BS & CS [4]; DS and ES [6].

**Epimer**	**8*R***	**8*R***	**8*R***	**8R**	**8R**
**Figure**	**AR**	**4**	**6**	**7**	**8**
C17	2.56, d (4),<br>2.74, d (4)	2.60, brd (3.89)<br>2.82, dd (1.97, 3.89)	2.53, d (4.34)<br>2.76, dd (1.87, 4.34)	2.56, d (4.3)<br>2.7, dd (1.8, 4.3)	2.55, d (4.3)<br>2.7, dd (1.8, 4.3)
Epimer	8S	8S	8S	8S	-
Figure	BS	CS	DS	ES	-
C17	2.27, d (3.5)<br>2.42, d (3.5)	2.29, d (3.9)<br>2.43, d (3.9)	2.3, d (3.8)<br>2.7, d (3.8)	2.2, d (3.8)<br>2.58, d (3.8)	-<br>-

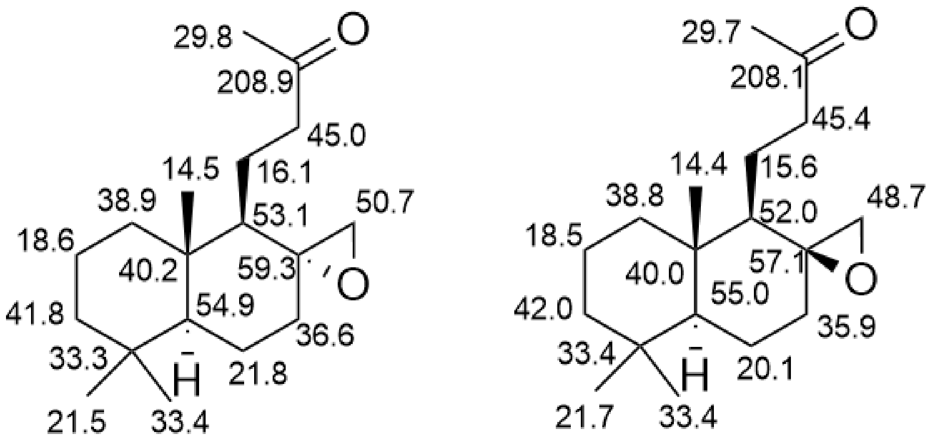

Figure S1. Standard chemical shifts for 8-spiroepoxy labdanes in the *R* and *S* configurations, as proposed by Bastard et al., [2].

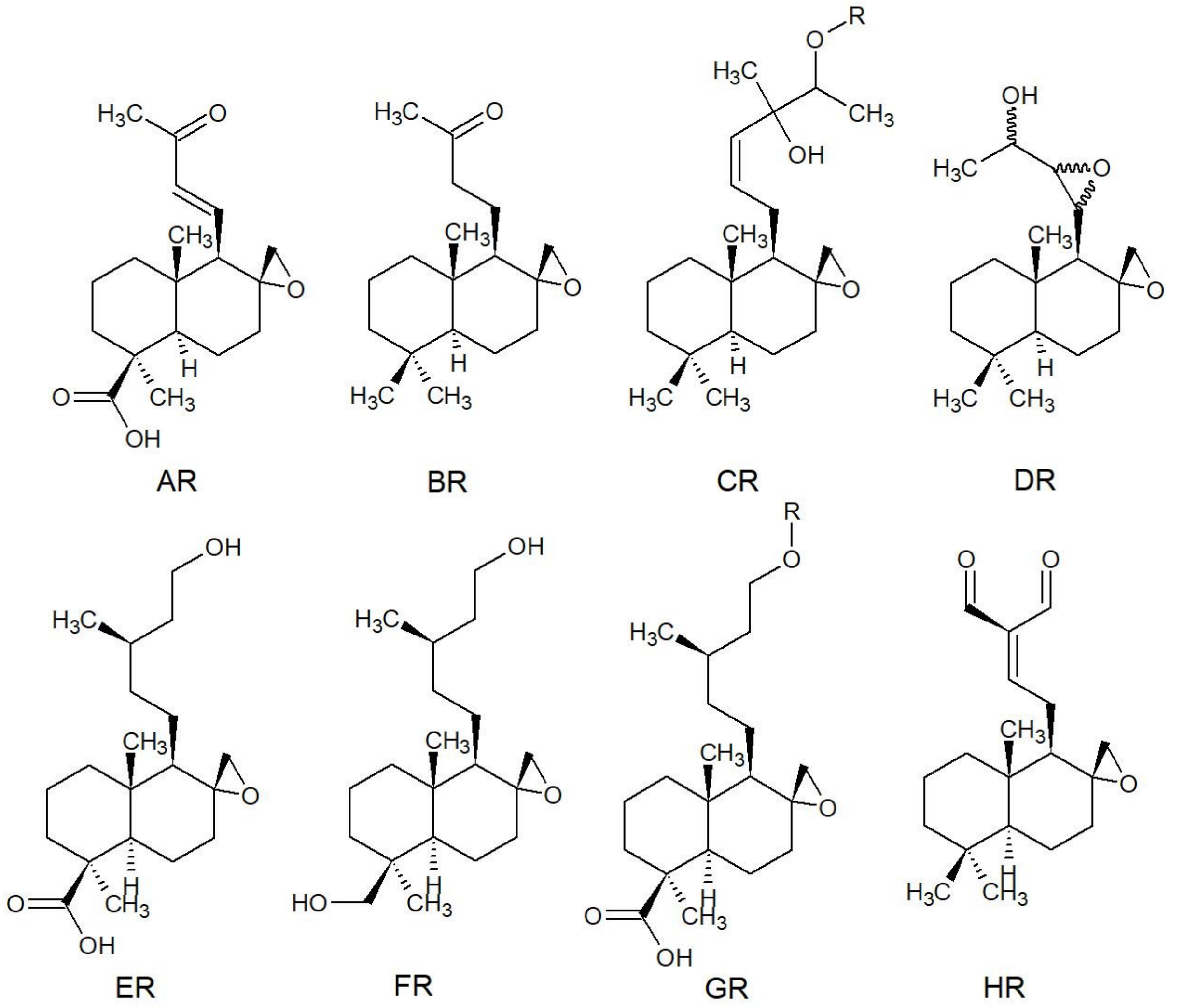

Figure S2. Labdanes with 8*R*-spiroepoxy conformation.

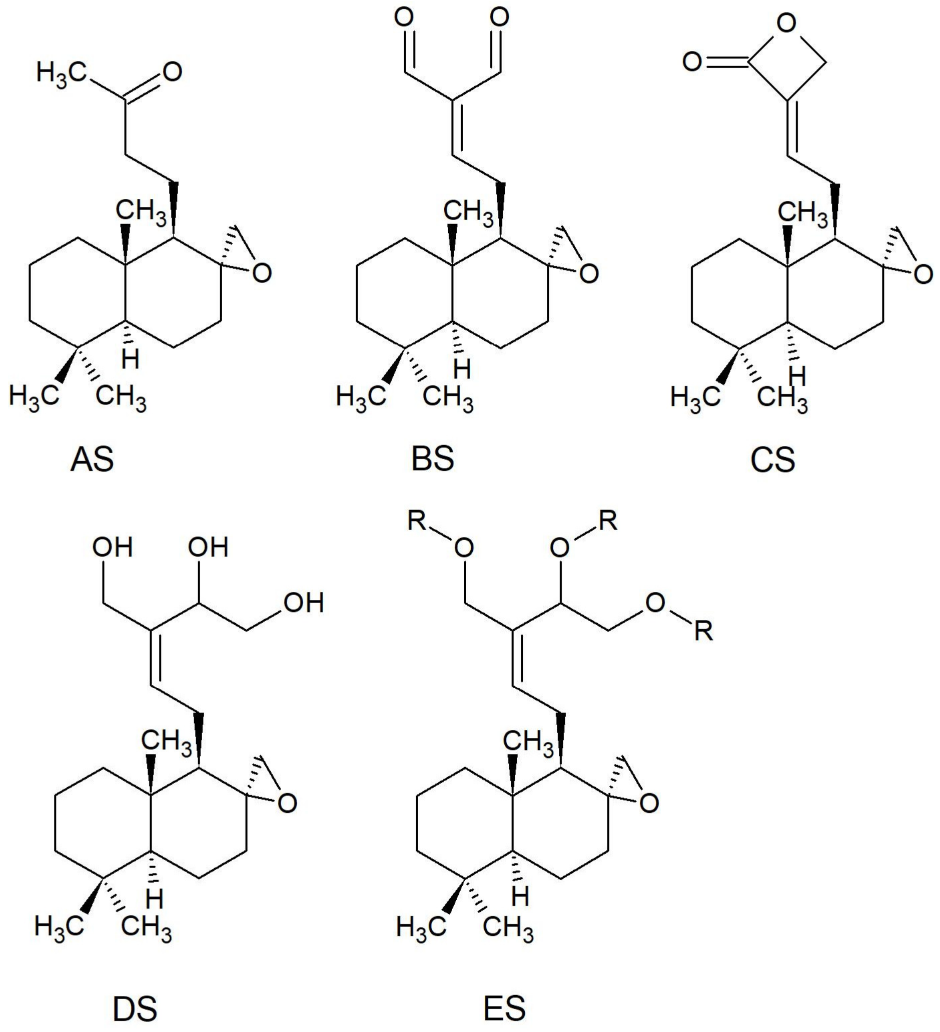

Figure S3. Labdanes with 8*S*-spiroepoxy conformation.

References

1. Fang, J.-M.; Sou, Y.-C.; Chiu, Y.-H.; Cheng, Y.-S. Diterpenes from the bark of *juniperus chinensis*. *Phytochemistry* **1993**, *34*, 1581-1584.
2. Bastard, J.; Duc, D.K.; Fetizon, M.; Francis, M.J.; Grant, P.K.; Weavers, R.T.; Kaneko, C. Cmr spectroscopy of labdanic diterpenes and related substances. *Journal of Natural Products* **1984**, *47*, 592-599.
3. Barrero, A.F.; Altarejos, J. 13c nmr data for labdane diterpenes. *Magnetic Resonance in Chemistry* **1993**, *31*, 299-308.
4. Schmeda-Hirschmann, G.; Astudillo, L.; Sepulveda, B.; Rodriguez, J.A.; Theoduloz, C.; Yanez, T.; Palenzuela, J.A. Gastroprotective effect and cytotoxicity of natural and semisynthetic labdane diterpenes from *araucaria araucana* resin. *Zeitzchrift fur Naturforschung. C, Journal of biosciences* **2005**, *60c*, 511-522.
5. Morita, H.; Itokawa, H. Cytotoxic and antifungal diterpenes from the seeds of *alpinia galanga*. *Planta Medica* **1987**, *54*, 117-120.
6. Sob, S.V.T.; Tane, P.; Ngadjui, B.T.; Connoly, J.D.; Ma, D. Trypanocidal labdane diterpenoids from teh seeds of *aframomum aulacocarpos* (zingiberaceae). *Tetrahedron* **2007**, *63*, 8993-8998.
